# Supplementary material for: Proposed Diagnostic Criteria for Invasive Pulmonary Aspergillosis in Patients with Autoimmune Inflammatory Rheumatic Diseases: A Proof-of-Concept Study
Source: J Fungi (Basel). 2025 Jun 7;11(6):437. doi: 10.3390/jof11060437 (PMC12194539; doi:10.3390/jof11060437)
Supplement: Supplementary file 1 [file jof-11-00437-s001.zip › jof-3667332-supplementary.pdf]

Table S1. Comparison of the IPA diagnostic/classification criteria

	**EORTC/MSGERC 2019**	**ICU (AJRCCM 2011)**	**COPD (ERJ 2007)**	**Our study**
Target patients	Patients with hematologic malignancies	ICU patients	Patients with COPD	Patients with AIIRDs
Host factors	Either of the following:<br>- PSL > 0.3 mg/kg/day<br>- T/B cell-targeted agents	Not required in case of culture positivity or microscopical detection	Both of the following:<br>- Any steroid therapy (any dose is acceptable, not required for proven IPA)<br>- Severe COPD	Any immunosuppressive therapy
Clinical features (CT)	Need typical CT findings	Any abnormality	- Any abnormality not responding to antibiotics	- Any abnormality not responding to antibiotics or immunosuppressive therapy
Clinical features (symptoms/clinical course)	Not required	Either of the following:<br>- Symptoms not responding to antibiotics<br>- Hemoptysis<br>- Chest pain<br>- Dyspnea	Respiratory distress not responding to antibiotics	Symptoms not responding to antibiotics or immunosuppressive agents
Mycological evidence	Either of the following:<br>(if not, classified as possible IPA)<br>- Positive culture or microscopy detection<br>- Serum GMA ≥ 1.0	Need a positive culture (GMA-positive not allowed)	Two consecutive positive tests for culture or microscopy or GMA or antibody<br>(if not, classified as possible IPA)	Either of the following:<br>(not required if typical radiologic findings exist)<br>- Positive culture<br>- Microscopical detection<br>- Serum GMA ≥ 1.0

Abbreviations: AIIRD, autoimmune inflammatory rheumatic diseases; AJRCCM, American Journal of Respiratory and Critical Care Medicine; COPD, chronic obstructive pulmonary disease; CT, computed tomography; EORTC/MSGERC, European Organization for Research and Treatment

of Cancer/Mycosis Study Group Education and Research Consortium; ERJ, European Respiratory Journal; GMA, galactomannan antigen; ICU, intensive care unit; IPA, invasive pulmonary aspergillosis; PSL, prednisolone

Table S2. Relationships of duration from the start of observation to death and the causes of mortality

Duration of survival (weeks)	Proven/probable IPA	Potential IPA
1	**IPA**	
1	**IPA**	
2	**IPA**	
3		**IPA**
3		**IPA**
6		**IPA** or ILD
7		Primary disease (ILD)
10		Unknown
20	Pulmonary embolism	
21	**IPA**	
25		**IPA** or Pulmonary MAC
42		Unknown
69		Peritoneal cancer
79	**IPA**	
87	Primary disease (ILD)	
137		Unknown
163	Primary disease (ILD)	
172	Primary disease (ILD)	
175		Unknown
182	Unknown	
193	Unknown	
282	Lung cancer	
367		Unknown

Abbreviations: MAC, *Mycobacterium avium* complex; ILD, interstitial lung disease; IPA, invasive pulmonary aspergillosis

Table S3. Pathogens causing IPA and antifungal therapy in the proven/probable IPA and potential IPA groups

	**Proven/probable IPA**	**Potential IPA**	**p**
Total number	24 (100%)	29 (100%)	-
Pathogen			
Aspergillus fumigatus	18 (78.2%)	8 (27.6%)	<0.001*
Aspergillus niger	3 (12.5%)	3 (10.3%)	0.893
Unable to identify the species	1 (4.3%)	2 (6.9%)	0.836
Culture-negative	0 (0%)	14 (48.2%)	0.002
First-line therapy	22 (95.7%)	23 (79.3%)	0.192
Voriconazole	18 (78.2%)	11 (37.9%)	0.009
Micafungin	3 (13.0%)	6 (20.7%)	0.723
Itraconazole	1 (4.3%)	5 (17.2%)	0.313
Liposomal amphotericin B	0 (0%)	1 (3.4%)	0.907
Second-line therapy	8 (34.8%)	11 (37.9%)	0.956
Voriconazole	3 (13.0%)	7 (24.1%)	0.513
Micafungin	2 (8.7%)	3 (10.3%)	0.785
Itraconazole	2 (8.7%)	0 (0%)	0.372
Liposomal amphotericin B	0 (0%)	0 (0%)	-
Total treatment period (weeks)	83.6	152.4	0.524
Death	12 (52.2%)	12 (41.4%)	0.620

Categorical variables were presented as n (%) unless otherwise indicated. p-values were calculated between the two groups using the chi-squared test.

* p-values of <0.05 were considered to indicate statistical significance.

Abbreviation: IPA, invasive pulmonary aspergillosis

Table S4. Comparison of pathogens and anti-fungal treatment between survivors and deaths among patients with potential IPA

	**Survivors**	**Deaths**
Total number	19 (100%)	12 (100%)
Pathogen		
Aspergillus fumigatus	18 (78.2%)	8 (27.6%)
Aspergillus niger	3 (12.5%)	3 (10.3%)
Unable to identify the species	1 (4.3%)	2 (6.9%)
Culture-negative	0 (0%)	14 (48.2%)
Clinical laboratory tests		
Serum BDG (pg/mL)	0 (0-76.1)	10.0 (0-14.4)
Serum GMA	0.7 (0.2-1.3)	0.8 (0.4-1.2)
<0.5	8 (47.1%)	8 (66.7%)
0.5-0.9	2 (11.8%)	4 (33.3%)
≥1.0	2 (11.8%)	5 (41.7%)
Patterns of abnormal shadows (repeatable)		
Bilateral shadows	9 (52.9%)	8 (66.7%)
Multiple shadows	4 (23.5%)	6 (50.0%)
Nodule	5 (29.4%)	12 (100.0%)
Dense, well-circumscribed lesions	3 (17.6%)	5 (41.7%)
Air crescent sign	1 (5.9%)	2 (16.7%)
Cavity	3 (17.6%)	7 (58.3%)
Wedge-shaped and segmental or lobar consolidation	2 (11.8%)	1 (8.3%)
Pleural effusion	1 (5.9%)	2 (16.7%)
First-line therapy	22 (95.7%)	22 (75.9%)
Voriconazole	18 (78.2%)	11 (37.9%)
Micafungin	3 (13.0%)	6 (20.7%)
Itraconazole	1 (4.3%)	5 (17.2%)
Liposomal amphotericin B	0 (0%)	1 (3.4%)
Second-line therapy	8 (34.8%)	10 (34.5%)
Voriconazole	3 (13.0%)	7 (24.1%)
Micafungin	2 (8.7%)	2 (6.9%)
Itraconazole	2 (8.7%)	0 (0%)
Liposomal amphotericin B	0 (0%)	0 (0%)

Categorical variables were presented as n (%) unless otherwise indicated. Continuous variables were presented as the median (interquartile range).

Abbreviation: BDG,(1,3)-beta-D-glucan; GMA, galactomannan antigen; IPA, invasive pulmonary aspergillosis

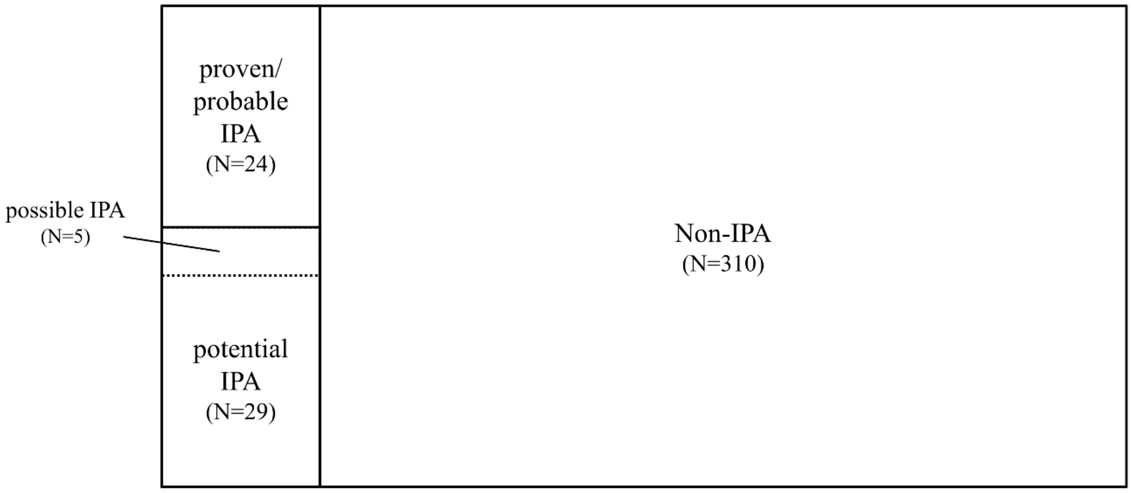

Figure S1. Schematic diagram of each classified group. The proven/probable IPA and possible IPA groups were classified according to the EORTC/MSGERC 2019 definition, and the potential IPA group was classified according to our proposed criteria.

Abbreviations: IPA, invasive pulmonary aspergillosis; EORTC/MSGERC, European Organization for Research and Treatment of Cancer/Mycosis Study Group Education and Research Consortium
